# Seasonal Comparison of Pre-Adolescent Soccer Players’ Physical Performance Using an Objective Physical Test Battery

**DOI:** 10.3390/jfmk9030166

**Published:** 2024-09-17

**Authors:** Giacomo Villa, Foivos Papaioannou, Manuela Galli, Veronica Cimolin

**Affiliations:** 1 Euleria srl Società Benefit, Via delle Zigherane, 4/A, 38068 Rovereto, TN, Italy; giacomo1.villa@polimi.it (G.V.); foivos@euleria.it (F.P.); 2 Department of Electronics, Information and Bioengineering (DEIB), Politecnico di Milano, 20133 Milano, MI, Italy; manuela.galli@polimi.it; 3 Istituto Auxologico Italiano, IRCCS, S. Giuseppe Hospital, Piancavallo, 28824 Oggebbio, VB, Italy

**Keywords:** soccer, seasonal variations, physical performance, pre–post study, test battery, wearable sensors, IMUs

## Abstract

Background/Objective: Soccer is a multifactorial sport, requiring physical, psychological, technical, and tactical skills to succeed. Monitoring and comparing physical characteristics over time is essential to assess players’ development, customize training, and prevent injury. The use of wearable sensors is essential to provide accurate and objective physical data. Methods: In this longitudinal study, 128 male adolescent soccer players (from Under 12 to Under 19) were evaluated at two time points (pre- and post-season). Participants completed the Euleria Lab test battery, including stability, countermovement and consecutive jumps, agility, and quick feet tests. A single Inertial Measurement Unit sensor provided quantitative data on fifteen performance metrics. Percentage changes were compared to the Smallest Worthwhile Changes to assess significant changes over time. Results: The results showed significant improvements in most variables, including a 19.7% increase in quick feet, 10.9% in stability, and 9.6% in countermovement jumps. In principal component analysis, we identified four principal components—*strength-power*, *balance*, *speed-agility*, and *stiffness*—that explained over 80% of the variance. Conclusions: These findings align with previous studies assessing seasonal changes in adolescent soccer players, showing that the proposed test battery seems to be adequate to highlight physical performance changes and provide coaches with meaningful data to customize training and reduce injury rates.

## 1. Introduction

Soccer is widely recognized as the most popular and practiced sport in the world [1,2,3]. It is defined as a dynamic and multifactorial sport, requiring players to possess well-developed physical, psychological, technical, and tactical skills to succeed [3,4,5]. The evaluation at different time points of these various domains and their comparison in time is crucial for monitoring players’ development, tailoring training programs, and reducing injury rates [6,7]. Additionally, the objective assessment of young soccer players facilitates the early identification of talented players, enabling them to access elite and tailored training programs [3,8]. Furthermore, data collection from healthy athletes allows for the creation of normative databases that can be used to assess whether injured players are ready for a safe return to sport [9].

Previous research has analyzed the extent to which the various talent factors (individually and in combination) have predictive power in relation to the future success of players [10]. Successful teams have been shown to outperform less successful teams in technical gestures such as pass completion, frequency of forward and total passes, and average touches per possession [11,12,13]. Nevertheless, prospective studies have acknowledged the crucial role of motor, physical and physiological abilities [11]. Specifically, muscular strength and power, speed, agility, jumping abilities, and balance are essential motor parameters for thoroughly evaluating the physical profile of soccer players, with these factors showing a significant correlation between motor test results and future success in youth soccer [3,10,14,15,16]. Associations in soccer also emphasize the importance of speed abilities (e.g., quick movements, acceleration or deceleration, and change in direction [17]) and technical skills (i.e., ball skills, including ball control, dribbling, and shooting [18]), highlighting the growing need to objectively assess the physical performance of young athletes [5,19,20,21]. Soccer players’ anthropometric measurements, physiological attributes, and physical abilities are typically evaluated using various quantitative test batteries [3,22,23]. Different test batteries can complement each other, and their combination can offer soccer coaches and athletic trainers a more comprehensive understanding of the characteristics necessary for success at the elite level [3,15,22].

Over the last few decades, a wide range of technological tools and software for the quantitative analysis of human performance during sports activities have been developed [19]. An alternative solution to the frequently chosen approach of motion capture systems for the assessment of human performance is wearable devices such as heart rate monitors, Inertial Measurement Units (IMUs), and Global Positioning System (GPS) or Local Position Measurement System (LPMS) trackers [20,24]. IMUs, in particular, have become widespread, especially since the advent of microelectromechanical system (MEMS) technology, which enabled highly miniaturized, relatively low-cost, and low-power sensors [25]. Previous studies have successfully used IMU-based wearable sensors to monitor athlete performance and reduce the risk of injury in various team sports such as volleyball [26,27], rugby [28], and baseball [29], as well as in individual activities like running [30], skiing [31], and tennis and other racquet sports [32,33]. The integration of accelerometers, gyroscopes, and magnetometers into IMUs allows for the measurement of raw accelerations and angular velocities during movements, enabling the estimation of temporal, kinematic, and dynamic parameters [21,25]. The integration of IMUs in physical test batteries produces important quantitative information that coaches and sports scientists can use to obtain individual athletes’ physical profiles, highlighting their strengths and weaknesses [21]. On the other hand, while providing helpful and objective information, the use of IMUs for athletes’ assessment could lead to extensive and difficult-to-interpret datasets [34].

In sports research, principal component analysis (PCA) is one of the most frequently employed statistical techniques to efficiently reduce the substantial amount of data collected into a smaller number of extracted factors [34]. PCA allows researchers to reduce data multicollinearity by identifying principal components (PCs) explaining a high percentage of the dataset variance, minimizing the loss of information [34]. Generally, coaches, trainers, and athletes tend to select the performance variables according to their professional experience and other athletes’ information (evidence-based or not), which risks excluding essential variables [35]. This justifies the need for objective methods to select these critical variables [34]. PCA has already been used to understand the behavior in different sports such as rugby [36,37], soccer [38], and basketball [39].

The aim of this study was to objectively assess the physical performance of pre-adolescent male soccer players over a competitive sports season, using a multi-test battery that integrates a single IMU sensor. It was hypothesized that the physical performance of the athletes tested would show a significant improvement over the season.

## 2. Materials and Methods

### 2.1. Participants and Design

This longitudinal study was designed to assess the physical performance of young soccer players and compare the results between pre- and post-season. The physical characteristics of the subjects were assessed at two time points (T0 = pre-season in the last week of August 2021 and T1 = post-season in the first week of May 2022), eight months apart.

A total of 128 male adolescent soccer players (age: 13.8 ± 2.1; height: 165.3 ± 13.4 cm; weight: 54.3 ± 13.1 kg; BMI: 19.5 ± 2.1 kg/m^2^) from the Under 12 to the Under 19 categories of the A.C. Trento 1921 youth sector were enrolled. Participation in the study was voluntary, and written informed consent was obtained from all participants and their parents or legal guardians. This study was conducted in accordance with the Declaration of Helsinki of 1964 and its latest amendments.

The inclusion criteria were as follows: (i) no injuries at the time of the evaluations or in the two weeks prior to the evaluations, (ii) no chronic lower limb conditions or diseases that could affect physical performance, and (iii) participation in at least 75% of soccer activities (i.e., training and soccer matches) between T0 and T1. This final inclusion criterion only applied to the T1 post-season assessment. Follow-up was completed for all participants, ensuring a 100% retention rate.

### 2.2. Materials

The physical test battery was performed using Euleria Lab (Euleria srl, Rovereto (TN), Italy) Class IIa (MDR 2017/745) medical software. The performance module integrated into Euleria Lab objectively assessed the physical performance using a position sensor integrated into a balance board and a single IMU. The IMU (Xsens DOT, Xsens Technologies B.V., Enschede, The Netherlands) is a small (36.30 × 30.35 × 10.80 mm) and lightweight (11.2 g) wearable sensor constituting of a 3D accelerometer (±16 g full-scale range), 3D gyroscope (±2000°/s full-scale range), and 3D magnetometer (±8 Gauss full-scale range). The data sampling rate used in this application was 60 Hz.

### 2.3. Data Collection

Before starting the physical test battery, height (cm) and weight (kg) were measured and registered for each participant. Height was measured to the nearest 0.1 cm using a stadiometer, and weight was measured to the nearest 0.1 kg using a calibrated analog scale (light indoor clothing, no shoes). Body mass index (BMI, kg/m^2^) was then calculated by dividing the body mass by the height (converted in m) squared. Moreover, each athlete’s dominant leg was determined by asking “If you had to throw a ball at a target, which leg would you use to throw the ball?”, according to van Melick et al. [40]. Of the 128 athletes included in this study, 109 (i.e., 85.2%) were classified as right-footed, and 19 (i.e., 14.8%) were classified as left-footed. All the tests were preceded by a supervised and standardized 10 min warm-up consisting of 6 min of jogging at the self-selected sub-maximal intensity and 4 min of low-intensity athletic drills, including lateral skip, high knee walk, vertical sub-maximal jumps, lateral and diagonal sub-maximal jumps, and change in directions. Sufficient recovery time of 3 min was allocated between each performance trial.

Given the non-grassy smooth surface on which the tests were conducted, participants were asked to wear sports shoes during the test session to avoid slipping and injuries. After two non-registered practice trials, a single attempt for each task was performed and registered. There was one minute of rest between two consecutive trials and five minutes of rest between two different tests.

The test battery included in the Euleria Lab medical software was performed in a systematic order consisting of the following five tests:

- Double-leg (DL-ST) and single-leg (SL-ST) stability tests performed both with the right (R-ST) and left (L-ST) foot;
- Double-leg (DL-CMJ) and single-leg (SL-CMJ) countermovement jump tests performed both with the right (R-CMJ) and left (L-CMJ) foot;
- Double-leg consecutive jump (CJ) test;
- Single-leg agility test, named parkour (PK) test;
- Quick feet (QF) test.

In the case of single-leg tests, the dominant leg was tested first. The time needed to perform the full test battery was about 30 min for each participant.

The test battery was designed by Hildebrandt et al. [9], who also determined its validity and reliability by using the intraclass correlation coefficient (ICC 1/1) in the one-way random-effect model.

#### 2.3.1. Stability Test

The stability test was performed on a balance board free to move in all directions (MFT Challenge Disc, TST—Trendsport Trading, Grosshöflein, Austria). A position sensor integrated into the board detects its inclination and provides feedback about the disc position in real time. The balance board was positioned at 75 cm from the screen on which a target with a blue dot representing the tilt of the disc was displayed. The height of the screen was also adjusted so that the target was at the athlete’s eye level [41]. Before starting the test, each participant was verbally instructed to keep the blue dot as close as possible to the center of the target for the whole test duration.

The test lasted 20 s for each configuration (i.e., DL-ST, R-ST, and L-ST). In the case of DL-ST, participants could place their feet at the distance they felt most comfortable with. In the case of SL-ST, the tested foot was positioned in the center of the disc, while the other foot was not allowed to touch the board or the ground to stabilize.

The variable obtained from the stability test is the level of stability (LoS, arbitrary units, a.u.) and can assume values between 1 and 5; it is calculated as follows:

(1) $$LoS\;(a.u.)=\frac{\sum_{i=1}^{5}i\times {samples}_{i-th\;zone}}{{samples}_{TOT}}$$

where i is the number of concentric circles in the target shown on the screen, and samples is the number of samples collected by the disc’s position sensor during the test. The closer the values obtained are to 1, the better the stability performance.

The test–retest reliability reported by Hildebrandt et al. [9] was moderate for the DL-ST (ICC = 0.688) and good for the SL-ST (ICC = 0.791 obtained by averaging the ICC values reported for R-ST and L-ST).

#### 2.3.2. Countermovement Jump Test

**Figure 1:**
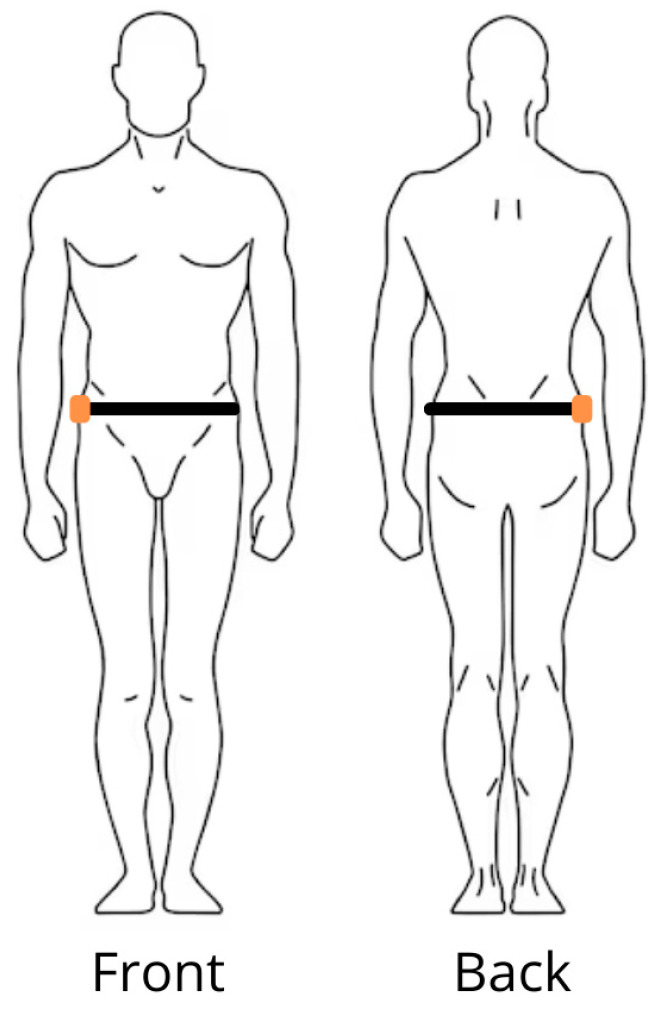
IMU sensor’s positioning (in orange) on the athlete’s flank through an elastic band (in black).

The CMJ test was performed with the participants wearing the IMU sensor at the flank level through an elastic band to minimize the sensor’s movement during jump execution (Figure 1).

In the case of the DL-CMJ, the sensor was placed on the dominant side, while in the case of the SL-CMJ, it was placed on the tested side.

Each participant was instructed on how to perform the CMJ correctly. The athletes were instructed to start from an upright position with their feet shoulder-width apart and to make a quick downward movement, bending at the hips and knees (countermovement phase). The countermovement phase had to be followed by an immediate and powerful upward movement (propulsive phase) and the landing phase. Participants were asked to keep their hands on their hips throughout the CMJ to minimize the effect of arm swing on jump performance, without touching the sensor so as not to interfere with the measurements. For the SL-CMJ, participants were asked to perform the counter movement and propulsive phases using only the tested leg but were allowed to land with both feet to minimize the risk of injury.

The variables calculated through the data collected by the IMU were the vertical jump height (CMJ_VJH_, cm) and the power developed during the jump (CMJ_POW_, W/kg) normalized by the subject’s body mass; they were calculated as follows [42]:

(2) $${CMJ}_{VJH}\left(cm\right)=\frac{{FT}^{2}\times g}{8}\times 100$$

(3) $${CMJ}_{Pow}(W/kg)=\frac{60.7\times {CMJ}_{VJH}+45.3\times body\;mass-2055}{body\;mass}$$

where FT is the flight time (s) corresponding to the time between take-off (TO) and landing (LA) instants, and g is the gravity acceleration (m/s^2^).

**Figure 2:**
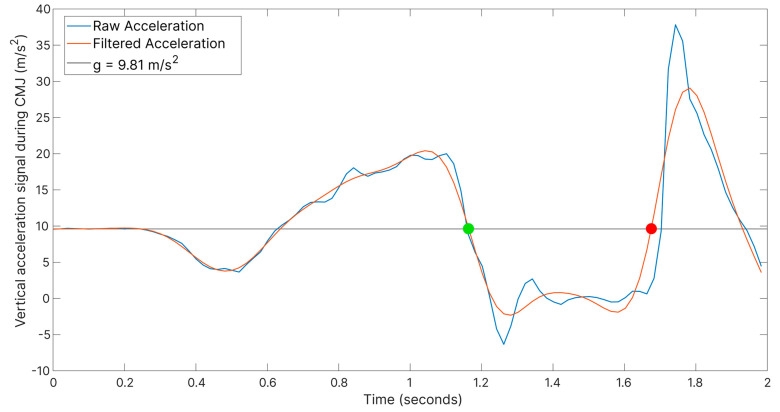
Vertical acceleration signal from a CMJ. TO (in green) was identified as the instant when filtered acceleration becomes <g, while LA (in red) was identified as the last instant when filtered acceleration is <g.

TO and LA instants were determined by analyzing the vertical acceleration of the IMU sensor filtered with a low-pass Butterworth filter (order = 3, cut-off frequency = 5 Hz). According to Nielsen et al. [43], TO is defined as the instant when the filtered acceleration becomes less than g, while LA is identified as the last observation of the filtered acceleration less than g (Figure 2).

The test–retest reliability reported by Hildebrandt et al. [9] was high for the CMJ_VJH_ and the CMJ_POW_ in the DL-CMJ (ICC = 0.921 and ICC = 0.889, respectively) and good for the CMJ_VJH_ and the CMJ_POW_ in the SL-CMJ (ICC = 0.838 and ICC = 0.818, respectively, obtained by averaging the ICC values reported for R-ST and L-ST).

#### 2.3.3. Consecutive Jump Test

The CJ test was performed with the participants wearing the IMU sensor at the flank level of the dominant side through an elastic band to minimize the sensor’s movement during the jumps (Figure 1).

Each participant was instructed on how to perform the CJ test correctly. Starting from the same position as for the CMJ, they were asked to perform five consecutive vertical jumps with the aim of maximizing the jump height with the shortest contact time possible between two consecutive jumps. Participants were allowed to use their arms during the jump execution.

The variables automatically calculated by the Euleria Lab software were the maximum vertical jump height (CJ_VJH_, cm), calculated using Equation (2), and the minimum contact time (CJ_CT_, ms), calculated as the time interval between two consecutive jumps. Moreover, a stiffness parameter (CJ_ST_, a.u.) was estimated as the ratio between the maximum vertical jump height and the minimum contact time and then converted to a.u. by multiplying it by the conversion factor (*10). The test–retest reliability reported by Hildebrandt et al. for the CJ_ST_ was high (ICC = 0.838) [9].

#### 2.3.4. Agility Test

**Figure 3:**
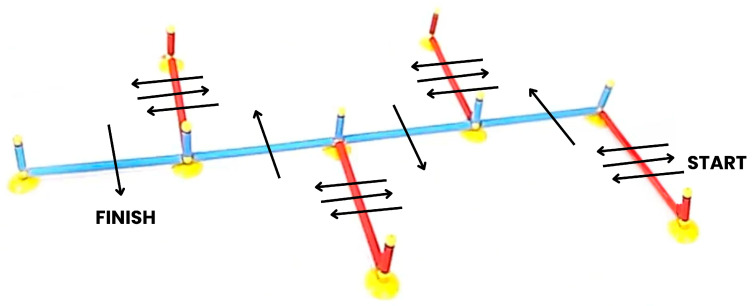
Hurdles’ positioning and jump coordination path to be followed for the agility test [9].

The agility test was performed by creating a jumping coordination path using the Speedy Basic Jump Set (TST Trendsport, Grosshöflein, Austria). Eight flexible hurdles 50 cm long were positioned horizontally on a flat surface and connected by plastic connectors attached to nine 10 cm vertical hurdles, as shown in Figure 3. The final dimensions of the coordination path were 2 m long, 1 m wide, and less than 5 cm high.

Participants wore the IMU sensor at the flank level of the dominant side through an elastic band, which minimized the sensor’s movement during the test. Each participant was instructed to correctly perform the test by following the predefined jump path, consisting of single-leg forward, backward, and lateral jumps, as shown in Figure 3. Double jumps without overcoming an obstacle, twisting the hip, or touching the ground with the raised leg were not allowed. The dominant side was tested first, followed by the non-dominant side.

The variable obtained from the PK test was the time (in seconds) required to complete the jump path, which was automatically measured by the IMU sensor through the automatic detection of the first and last jump in the sequence.

The test–retest reliability reported by Hildebrandt et al. [9] for the PK test was high (ICC = 0.809 obtained by averaging the ICC values reported for R-ST and L-ST).

#### 2.3.5. Quick Feet Test

**Figure 4:**
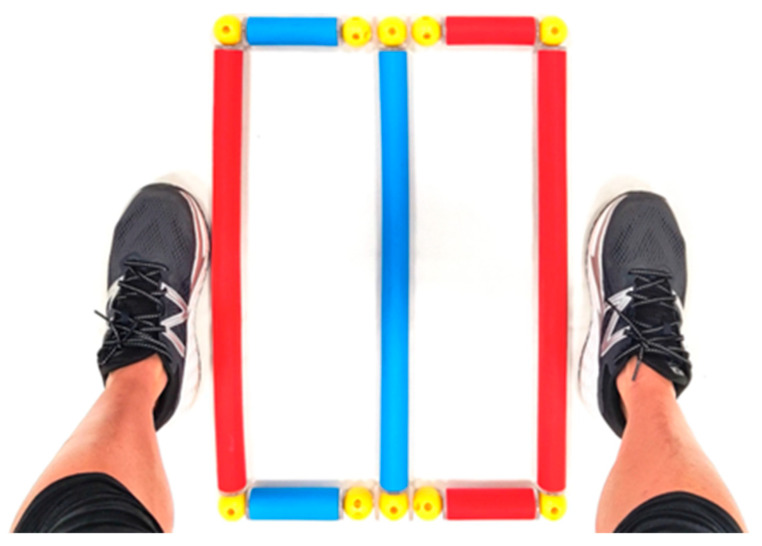
Hurdles’ positioning to perform the QF test [9].

The QF test was performed by positioning the hurdles of the Speedy Basic Jump Set (TST Trendsport, Grosshöflein, Austria) to create an obstacle, as shown in Figure 4.

Participants wore the IMU sensor in the sagittal plane on the mid-thigh of the dominant side through an elastic band, which minimized the sensor’s movement during the test. Each participant was instructed to perform the test by stepping in and out from the obstacle until the completion of 15 repetitions. One repetition was finished when the starting leg returned to its initial position.

The variable obtained from the PK test was the time (in seconds) required to complete the 15 repetitions automatically measured by the IMU sensor through the automatic counting of the repetitions. The test–retest reliability reported by Hildebrandt et al. [9] for the QF test was high (ICC = 0.803).

### 2.4. Bias

Efforts to minimize bias risk included the utilization of the same equipment and experimental protocol for all the measurements at both T0 and T1 time points. All the assessments were performed by trained personnel.

### 2.5. Statistical Analysis

Data were tested using parametric statistical analysis following a preliminary checking for normality and homogeneity of variances (using the Shapiro–Wilk’s and Levene’s tests, respectively). Descriptive statistics for each variable were calculated and expressed as mean ± standard deviation (SD). Eventual asymmetries in single-leg tests (i.e., CMJ and PK tests) were assessed through a paired samples *t*-test (with α < 0.05).

To highlight changes in performance between time points, percentage change (%C) was calculated for all variables using Equation (4) [44].

(4) $$\%C\;\left(\%\right)=\frac{{mean}_{T0}-{mean}_{T1}}{{mean}_{T0}}\times (-100)$$

where ${mean}_{T}$ is the mean value calculated at the corresponding time point.

To assess whether the differences between the time points were statistically significant, the %C values were compared with the smallest worthwhile change (SWC) expressed as a percentage [44]. The SWC can be calculated by multiplying the test SD by 0.2, based on Cohen’s effect size principle, where 0.2 represents a small but not trivial effect size [44]. The formula for calculating the SWC is therefore

(5) $$SWC=\frac{SD\times 0.2\;}{{mean}_{T0}}\times 100$$

and interpreted by the following: if the absolute value of the %C is greater than the SWC, then the difference is statistically significant. Otherwise, the difference is not statistically significant [44].

Lastly, PCA was performed to reduce the dimensionality of the dataset by composing correlated variables while retaining as much of the dataset variability as possible. Variables within a given PC are correlated with each other, while the PCs themselves do not correlate and thus explain distinct information. Before conducting the PCA, all the data were centered and scaled [45]. The Kaiser–Meyer–Olkin (KMO) test was used to verify the sampling adequacy of the data, with a value of 0.5 used as a threshold for acceptability [46]. Bartlett’s test of sphericity was also used to determine the suitability of the data for PCA, with significance accepted at an α level of *p* ≤ 0.05. Orthogonal rotation (varimax) was used to improve the identification and interpretation of the factors [34,47]. Therefore, following the multi-faceted approach recommended by Hair et al. [47], the optimal number of principal components (PCs) to extract and interpret was determined. This approach involved examining the scree plot, analyzing eigenvalues, and assessing the cumulative variance explained by each PC [47].

Statistical and data analyses were conducted using JASP (JASP Team 2023, Version 0.17.3) and MATLAB (v.2023a MathWorks, Natick, MA, USA).

## 3. Results

A total of 15 variables from 128 subjects were collected and analyzed at two time points (T0 and T1) (Table 1).

The paired samples *t*-test showed no statistical differences between the right and left sides in the CMJ parameters (CMJ_VJH_ and CMJ_POW_) and the PK test, at both time points.

%C values with a negative sign indicate that the reported value is higher at T0 than at T1. The smallest %C was found for the contact time in the CJ, which improved by 2.4% and is the only variable showing a non-significant difference between the two time points (%C < SWC). Variables such as DL-CMJ_VJH_, DL-CMJ_POW_, and PK showed %C values of around 6%. Although statistically significant, these %C values are close to the SWC value. The variables showing the highest %C between the two time points were the time in the QF test (absolute %C = 17.8%), the power in the single-leg CMJ (mean %C = 13.1%) and the stiffness in the CJ (%C = 12.5%).

According to the KMO test, two variables (CJ_CT_ and CJ_ST_) had measure of sampling adequacy (MSA) values lower than the acceptability threshold (MSA < 0.5). For this reason, CJ_CT_ and CJ_ST_ were discarded for further analysis. The overall MSA was 0.694 and 0.663 for the T0 and T1 datasets, respectively. Barlett’s test of sphericity confirmed the suitability of the data (*p* < 0.001) for both datasets.

**Figure 5:**
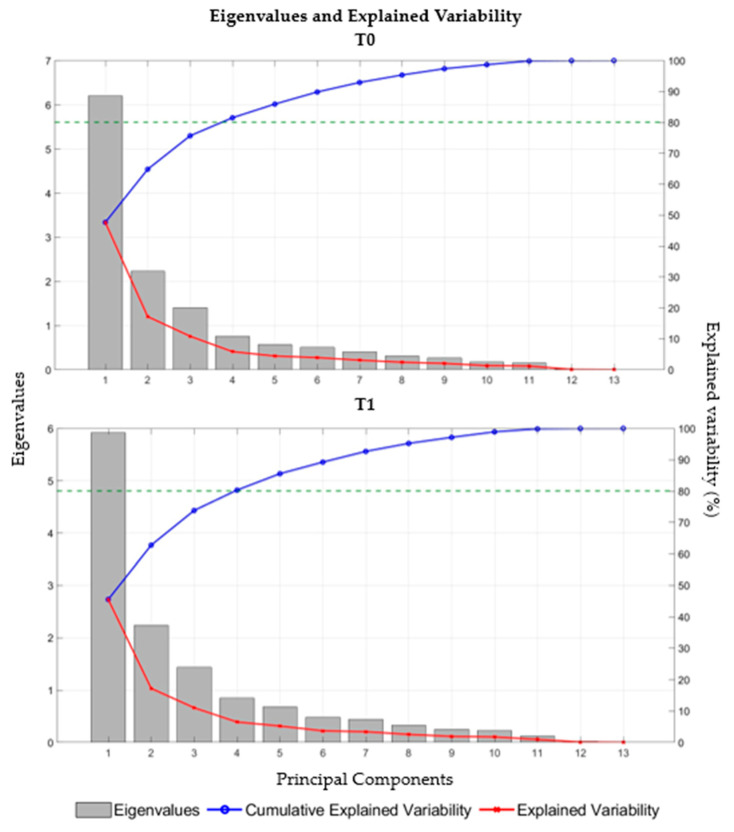
Eigenvalues, cumulative explained variability (in %), and explained variability (in %) at T0 (**top**) and T1 (**bottom**) for each PC. The dashed green line corresponds to the 80% threshold of explained variability.

Figure 5 shows the weight of each eigenvalue, the cumulative explained variability, and the explained variability at the two time points (T0 and T1). In accordance with the recommendations suggested by Hair et al. [47], the first four PCs were extracted for both T0 and T1 datasets. Numerical values of eigenvalues, the variability explained, and the cumulative variability explained for the first four PCs in the two time points are reported in Table 2.

Table 3 and Table 4 display the component loadings with a coefficient higher than 0.4 of the variables to the first four PCs at the T0 and T1 time points, respectively.

Table 3 and Table 4 report that PC1 contains variables afferent to the CMJ parameters (both VJH and power developed), and PC2 groups the LoS parameters both from the DL and SL stability tests, while PC3 comprises the variables from the PK and QF tests. The last component (PC4) consists only of the CJ_VJH_ variable.

## 4. Discussion

This longitudinal study had the aim to objectively assess the physical performance of 128 pre-adolescent male soccer players over a competitive season using a multi-test battery through a single IMU sensor. The test battery included stability, countermovement jump, consecutive jump, agility, and quick feet tests, and was designed to assess athletes’ performance in the areas of balance, agility, speed, and strength. The quantitative assessment of the physical performance at the two time points enables the identification of changes that have occurred during the sports season. The discrepancies were quantified through the calculation of the percentage change (%C) and the smallest worthwhile change (SWC).

Previous research has established a strong correlation between balance skills and the risk of lower limb ligament injuries in team sports such as soccer [48,49] and basketball [50], underscoring the importance of balance testing in evaluating athletes’ performance and injury risk [9]. The Euleria Lab test battery revealed a significant improvement in balance abilities over the course of the sports season, suggesting that the training programs were effective in enhancing balance skills. However, it is important to acknowledge the role of growth and maturation of athletes in performance improvements. Previous studies have emphasized the necessity of incorporating specific balance training into regular routines, as enhanced balance abilities are closely linked to improved technical skills [51]. Jumping abilities are another key success factor in sports like soccer, with studies showing a positive correlation between jumping performance and various motor skills [52,53]. Consistent with other research on seasonal changes in adolescent soccer players [54,55], CMJ performance improved throughout the season, demonstrating the effectiveness of the training programs in enhancing lower limb strength and power in pre-adolescent players. Nevertheless, the potential impact of biological maturation on these improvements should be considered. The assessment of asymmetries is also crucial for the monitoring of the risk of injury [9,52]. The absence of statistically significant differences in single-leg performance between the lower limbs at both time points suggests a general symmetry among the athletes. However, it remains essential to consider individual characteristics when developing personalized training programs aimed at reducing injury rates. Significant improvements were also observed in the quick feet test, indicating that the activities performed during the season contributed to enhanced agility and foot speed, according to similar studies [56,57].

Overall, the results of the present study demonstrate that the activities undertaken by the athletes during the sports season positively impacted their motor abilities and performance. The observed improvements can likely be attributed to the effective training programs designed by the soccer coaches and the competitive matches played throughout the season. Additionally, the biological maturation and growth of the athletes likely played a significant role in their enhanced physical performance. Moreover, the proposed test battery seems to be adequate for the physical assessment of adolescent soccer players. Nevertheless, as soccer a is multifactorial sport requiring players to possess well-developed physical, psychological, technical, and tactical skills, the presented test battery should be integrated with other tests to provide a more holistic view of the soccer players [3,4,5].

The use of PCA allowed us to condense the multivariate dataset into fewer components while retaining as much of the dataset variability as possible. In accordance with Hair et al. [47], four PCs explaining more than 80% of the variance were obtained. The first PC refers to the strength and power motor ability, the second PC can be associated with the balance motor ability, PC3 is correlated to speed and agility performance, and the last PC can be associated with stiffness ability. Although PCA is a widely used statistical method in sports science [34], the comparison of the results obtained in the present study with those of similar studies is complex due to the significant differences in the type of metrics analyzed and specific objectives. Nevertheless, the PCA conducted in a similar study has revealed force, static and dynamic balance, and rapidity to be significant parameters for classifying soccer players in different levels of competition [58]. In the present study, the PCA enabled the identification of the consecutive jump test as the least influential component of the test battery. Consequently, if the practitioners wish to reduce the test battery, the consecutive jump test may be excluded from the battery without a significant loss of information.

### Limitations and Considerations

The outcomes of the present study might have been influenced by several factors. Firstly, carrying out the quantitative assessments at only two time points (i.e., at the beginning and at the end of the sports season) may not have been sufficient to clearly investigate the variations in athletes’ performance over the eight-month period. For this reason, an intermediate assessment would have provided additional information and a better understanding of the evolution of motor performance over time. In addition, it would be worthwhile to fully assess the long-term effects of training and athletes’ adaptations, as well as the growth dynamics of adolescent players. As a matter of fact, similar longitudinal studies have extended over several seasons to capture meaningful developmental changes in athletes’ physical capabilities [59].

Another limitation factor could lie in the absence of biological maturity estimation. Previous studies have shown that adolescents experience rapid physical and physiological changes during puberty, which may affect overall athletic performance (motor skills, strength development) [14] and increase the risk of injury [60]. Various methods for assessing and estimating the biological maturity of athletes have been identified and validated in the literature [61,62,63,64]. As differences in biological maturity between participants may lead to differences in baseline performance levels and responses to training stimuli, further studies should be undertaken to quantify the effect of biological maturity in soccer players on physical performance.

Moreover, individual variability in training adherence also plays a critical role in shaping performance outcomes. Although this study included only those athletes who participated in at least 75% of soccer-related activities (training and matches), commitment during training can vary widely due to differences in motivation, commitment, and external obligations such as academic workload or personal circumstances. This variability can significantly impact the extent of physiological adaptations achieved during the study period. Further studies should also consider the variability associated with the different athletic training schedules to which athletes have been subjected and consider how this variability impacts athletes’ performance. Furthermore, external factors such as environmental conditions (e.g., weather, playing surface), competitive schedules, and coaching methods contribute substantially to performance outcomes and athletes’ physical responses. These factors introduce variability that may not be fully controllable, potentially affecting the consistency and magnitude of the performance changes observed across participants.

Finally, some methodological limitations could be identified. Firstly, despite numerous researchers placing the IMU during CMJs at the level of the fifth lumbar vertebra (L5) [65], the Euleria Lab software advocates sensor placement on the athletes’ flank. A previous study investigating how IMU positioning—on the flank or on the lumbar region—affects vertical jump height estimates showed limited differences between the two different positions [66]. Moreover, the calculation of the parameters in the consecutive jumps could be improved. Currently, the vertical jump height parameter is obtained as the maximum vertical jump achieved by the athlete over the five jumps performed. A better way of assessing the vertical jump height should be to calculate the average of the three central jumps out of the five performed, excluding the first and the last as they are significantly different from the others. The same adjustment should be made for the contact time. The calculation of the muscular stiffness as a ratio between the two will also be influenced by this adjustment.

## 5. Conclusions

In conclusion, this study utilized wearable sensors and statistical methods to assess the physical performance of pre-adolescent soccer players over a competitive sports season. The use of Euleria Lab (Euleria srl, Rovereto (TN), Italy) test battery integrating an IMU sensor provided a robust framework for objective evaluation across various physical domains critical for football. Moreover, it can be used in other contexts for the assessment of athletes from different sports backgrounds and can be performed without any space or movement constraints. The Euleria Lab system is an advantageous alternative compared to other technologies (i.e., optoelectronic systems and force platforms) as it is cheaper, more compact, and transportable, and it does not require long procedures to prepare the test or extract the data. In accordance with previous studies, the test battery allowed us to observe significant changes across variables assessing stability, strength, speed, and agility domains between pre-season and post-season. Nevertheless, given the multifactorial nature of football, the test battery should be integrated with other tests assessing the psychological, technical, and tactical skills of the football players.

From a clinical point of view, the results obtained could be provided to coaches and trainers to extract crucial information for the effective development of the athletes. These findings contribute indeed to the evolving understanding of athletic development and underscore the importance of tailored training strategies to optimize youth football performance and reduce the risk of injuries.

Future research should consider increasing the assessment time points to provide a clearer inspection of athletes’ variation over time and extend the follow-up periods to better capture developmental changes in young athletes’ physical abilities. Additionally, it is essential to assess and incorporate individual growth and biological maturity, along with the monitoring of training schedules and loads and external factors, to enhance the validity and applicability of findings in youth athlete development programs. Expanding the sample size and including diverse demographic groups would also improve the generalizability of the findings across different populations of young athletes.

## Figures and Tables

**Table 1:** Results (mean ± SD) of performance measures of the athletes at the two time points (*T*0 = pre-season; T1 = post-season).

Variables	T0	T1	%C	SWC
DL-LoS (a.u.)	3.1 ± 0.7	2.8 ± 0.7	**−9.7%**	4.4%
R-LoS (a.u.)	3.0 ± 0.6	2.7 ± 0.6	**−10.0%**	3.9%
L-LoS (a.u.)	3.1 ± 0.6	2.8 ± 0.6	**−9.7%**	3.8%
DL-CMJ_VJH_ (cm)	33.0 ± 6.1	35.0 ± 5.6	**6.1%**	3.6%
R-CMJ_VJH_ (cm)	19.0 ± 3.9	20.8 ± 4.3	**9.5%**	4.1%
L-CMJ_VJH_ (cm)	18.9 ± 4.3	21.3 ± 4.9	**12.7%**	4.5%
DL-CMJ_POW_ (W/kg)	43.1 ± 7.7	45.7 ± 6.5	**6.0%**	3.6%
R-CMJ_POW_ (W/kg)	26.8 ± 8.6	30.0 ± 7.8	**11.9%**	6.4%
L-CMJ_POW_ (W/kg)	26.7 ± 8.9	30.5 ± 8.0	**14.2%**	6.7%
CJ_VJH_ (cm)	28.0 ± 8.1	31.0 ± 8.2	**10.7%**	5.8%
CJ_CT_ (ms)	184.2 ± 50.9	179.7 ± 44.6	−2.4%	5.5%
CJ_ST_ (a.u.)	1.6 ± 0.5	1.8 ± 0.4	**12.5%**	5.8%
R-PK (s)	6.1 ± 1.3	5.7 ± 0.7	**−6.6%**	4.1%
L-PK (s)	6.2 ± 1.4	5.8 ± 0.8	**−6.5%**	4.5%
QF (s)	9.0 ± 2.0	7.4 ± 1.2	**−17.8%**	4.3%

Abbreviations: DL, double leg; R, right; L, left; LoS, level of stability; a.u., arbitrary units; CMJ, countermovement jump; VJH, vertical jump height; POW, power; CJ, consecutive jumps; CT, contact time; ST, stiffness; PK, parkour; QF, quick feet; %C, percentage change; SWC, smallest worthwhile change. Statistically significant %C values (absolute value %C > SWC) are shown in **bold**.

**Table 2:** Component characteristics (eigenvalue, explained variability, and cumulative variability) for the first four PC at the two time points, T0 and T1.

PC	T0			T1		
	Eigenvalue	Explained Var.	Cumulative Var.	Eigenvalue	Explained Var.	Cumulative Var.
PC1	6.197	0.477	47.7%	5.919	0.455	45.5%
PC2	2.232	0.172	64.8%	2.238	0.172	62.7%
PC3	1.407	0.108	75.7%	1.436	0.110	73.8%
PC4	0.759	0.058	81.5%	0.847	0.065	80.3%

Abbreviation: Var., variability.

**Table 3:** Component loadings of the variables to each PC at the T0 time point.

Var	PC1	PC2	PC3	PC4
DL-LoS		0.833		
R-LoS		0.860		
L-LoS		0.890		
DL-CMJ_VJH_	0.883			
R-CMJ_VJH_	0.839			
L-CMJ_VJH_	0.871			
DL-CMJ_POW_	0.901			
R-CMJ_POW_	0.847			
L-CMJ_POW_	0.877			
CJ_VJH_				0.931
R-PK			0.880	
L-PK			0.925	
QF			0.640	

Abbreviations: DL, double leg; R, right; L, left; LoS, level of stability; CMJ, countermovement jump; VJH, vertical jump height; POW, power; CJ, consecutive jumps; PK, parkour; QF, quick feet.

**Table 4:** Component loadings of the variables to each PC at the T1 time point.

Var	PC1	PC2	PC3	PC4
DL-LoS		0.729		
R-LoS		0.894		
L-LoS		0.904		
DL-CMJ_VJH_	0.842			
R-CMJ_VJH_	0.881			
L-CMJ_VJH_	0.870			
DL-CMJ_POW_	0.837			
R-CMJ_POW_	0.872			
L-CMJ_POW_	0.893			
CJ_VJH_				0.865
R-PK			0.870	
L-PK			0.851	
QF			0.635	

Abbreviations: DL, double leg; R, right; L, left; LoS, level of stability; CMJ, countermovement jump; VJH, vertical jump height; POW, power; CJ, consecutive jumps; PK, parkour; QF, quick feet.

## Data Availability

The raw data supporting the conclusions of this article will be made available by the authors upon request.
